# TAVI and Post Procedural Cardiac Conduction Abnormalities

**DOI:** 10.3389/fcvm.2018.00085

**Published:** 2018-07-03

**Authors:** Antonio Mangieri, Claudio Montalto, Matteo Pagnesi, Giuseppe Lanzillo, Ozan Demir, Luca Testa, Antonio Colombo, Azeem Latib

**Affiliations:** ^1^IRCCS San Raffaele Scientific Institute, Milan, Italy; ^2^Department of Cardiology, IRCCS Policlinico San Donato, Milan, Italy

**Keywords:** transcatheter aortic valve implantation, pacemaker, left bundle branch block, right bundle branch block, aortic stenosis

## Abstract

Transcatheter aortic valve implantation (TAVI) is a worldwide accepted alternative for treating patients at intermediate or high risk for surgery. In recent years, the rate of complications has markedly decreased except for new-onset atrioventricular and intraventricular conduction block that remains the most common complication after TAVI. Although procedural, clinical, and electrocardiographic predisposing factors have been identified as predictors of conduction disturbances, new strategies are needed to avoid such complications, particularly in the current TAVI era that is moving quickly toward the percutaneous treatment of low-risk patients. In this article, we will review the incidence, predictive factors, and clinical implications of conduction disturbances after TAVI.

## Introduction

As transcatheter aortic valve implantation (TAVI) evolves toward treating patients with lower surgical risk and greater life expectancy (1), a significant effort should be directed at a better understanding of common complications following this procedure.

New-onset conduction disturbances are common after TAVI, occurring in as much as 34.8% of patients at hospital discharge (2), and with left bundle branch block (LBBB) being the most common significant conduction disturbance after TAVI (10.5%) (2, 3). Although many studies investigated this topic, indications for permanent pacemaker implantation (PPI) are still unclear, often resulting in overtreatment.

The aims of the present review are to elucidate the anatomical and pathophysiological basis of these complications, to systematically illustrate currently available data, and to highlight unclear areas that clinical research still need to unveil.

## Anatomy and pathophysiology

A high incidence of conduction disturbances occurs not only following TAVI, but also after surgical aortic valve replacement (4), mainly because of the close anatomical relationship between the aortic valve and fundamental structures of the heart conduction system. The atrioventricular (AV) node lies within the apex of the triangle of *Koch*, at the convergence of the tendon of *Todaro* and of the attachment of the tricuspid septal leaflet in the right atrium. It continues as the bundle of His, piercing the membranous septum and penetrating through the central fibrous body to the left. Three major variants of AV nodes have been described, with 50% of individuals exhibiting a relatively right-sided AV bundle and 30% with a left-sided AV bundle, whereas in about 20% of patients the bundle courses under the membranous septum just below the endocardium (5). The last 2 above-described variants may expose patients to a higher risk of TAVI-induced conduction disturbances, especially in patients with a short membranous septum (5).

**Figure 1:**
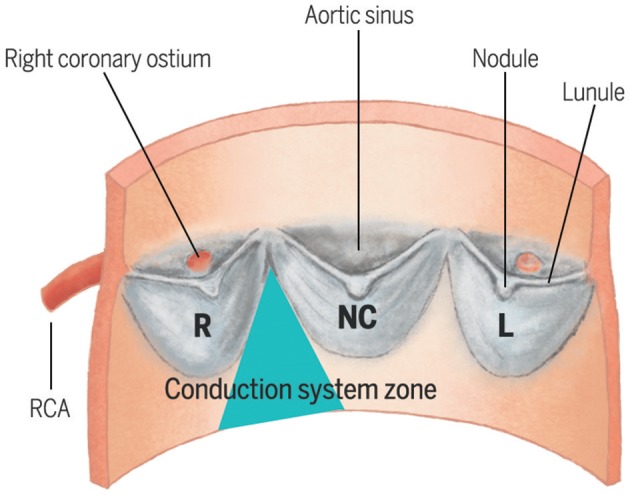
Spatial relationship between the three cusps of the aortic valve and the zone where the left bundle branch emerges beneath the membranous septum. L, Left cusp; NC, Non-Coronary cusp; RCA, Right Coronary Artery.

The left bundle branch emerges immediately beneath the membranous septum and is positioned superficially on the crest of the interventricular septum, and is intimately related to the base of the interleaflet triangle separating the non-coronary and right coronary leaflets of the aortic valve (3) (Figure 1). Consequently, when operating on the aortic valve, the risk exists to mechanically damage the nearby conductive system. TAVI may acutely expose the conduction system to an ischemic and inflammatory damage, in conjunction with a subacute process of healing (6), which may account for later and overall rarer conduction disturbances. Technical aspects of TAVI procedures, especially self- vs. balloon-expandable valve deployment system (7) and depth of implantation (8), are major factors in directly determining this acute mechanical damage to the conduction system. Furthermore, especially when treating intermediate-risk patients with greater life expectancy, a balance might exist between higher pre- and post-dilation pressures, needed to reduce paravalvular leak and the risk of a direct mechanical damage to the conduction system.

Finally, the close anatomical relationship with the aortic valve could also account for a certain degree of senile calcium deposition on the conduction system, which has been associated with the occurrence of LBBB and advanced atrioventricular block (AVB) in patients with aortic stenosis (9).

## Left bundle branch block

Overall the most common conduction alteration post-TAVI is new-onset LBBB (8), whose timing varies consistently and reflects different entities and reversibility of damage to the conduction system (Table 1); results of studies are summarized in Table 2. For the sake of clarity, we will refer to *new onset LBBB* as all LBBB which developed after TAVI, to *persistent LBBB* as all those who did not resolve at the time of discharge, while those patients who did not present LBBB will be referred to as *LBBB-free*.

**Table 1:** Timing of new-onset conduction abnormalities after TAVI.

	**Onset**	**Proposed mechanisms**	**Incidence**	**Resolution**	**Clinical implication**	**References**
LBBB	Intraprocedural (before valve implantation)	Guide wire insertion and balloon pre-dilation	46.5%			(10)
	Post-procedural (early)	Acute mechanical injury (ischemia, inflammation)	10.5–28.2%	At hospital discharge: 18.1% self-resolves; 40.1–57.4% persists and 11.5 evolves toward complete AVB	The most frequent occurrence of LBBB after TAVI	(2, 6–8, 10)
	Post-procedural (late)		2–6.2%	At long-term follow-up: 57.4% self-resolves; 14.8% persists and 18% evolves toward complete AVB		
				With self-expandable TAVI a higher rate of persistent LBBB (45%) was observed at follow-up.		
	At follow-up	Subacute damage (ischemia, healing)	0–2.9%		This represents a rare phenomenon	

TAVI, Transcatheter Aortic Valve Implantation; LBBB, left bundle branch block; AVB:

**Table 2:** Evidences on the clinical impact of LBBB after TAVI.

**References**	**N**	**TAVI type**	**New onset LBBB, n (%)**	**Results**	**Other**
(2)	1151	Balloon-expandable	121 (10.5)	No difference in mortality (both overall and heart-related) at 30-days and 1-year follow-up Higher PPI in LBBB group at 1-year follow-up (*p* = 0.001) Lower EF in the LBBB group at 1-year follow-up (53.4% vs. 57.4%; *p* = 0.02)	All patients were included in the PARTNER trial; ^†^
(11)	202	Balloon-expandable	61 (30.2)	No difference in mortality at 1-year follow-up No EF recovery at 1-year follow-up (53 ± 13% vs. 62 ± 9%; *p* = 0.0014) in the persistent LBBB group Higher PPI in LBBB group (34.2 vs. 4.3%; *p* = 0.001) No sudden death in patients with persistent LBBB and no PPI at discharge, but higher rates of syncope (16.0 vs. 0.7%; *p* = 0.001) and need for PPI (20.0 vs. 0.7%; *p* = 0.001) at 1-year follow-up Worse NYHA class at 1-year follow-up (*p* = 0.034)	^†^
(12)	668	Balloon-expandable	128 (19.2)	No difference in mortality at 13-month follow-up, even after stratifying for several risk factors; a landmark analysis at 30-d confirmed this finding No association at 13-month follow-up with rehospitalization, both for all causes and for heart failure Worse NYHA functional class at 6- month and 1-year follow-up (*p* = 0.015) No EF recovery (55% vs. 60%; *p* = 0.014) at 13- month follow-up	Four participating centers; ^†^
(13)	92	Self-expandable	34 (37)	No difference in mortality and/or rehospitalization at 1-year follow-up Higher PPI in LBBB who developed complete AVB No EF recovery at 6-month follow-up (Δ = 7.39 ± 9.05% vs. −0.46 ± 5.63%, *p* = 0.0001) No reverse remodeling at 1-year follow up (ESV 54.5 mL vs. 46 mL, *p* < 0.05; EDV 104 mL vs. 89 mL, *p* < 0.05)	^†^
(14)	27	Self-expandable	14 (52)	EF decreased after TAVI in patients with new conduction abnormalities (47 ± 12% to 44 ± 10% vs. 49 ± 12% to 54 ± 12%) No data on follow-up available	Patients with previous conduction disturbances were included in the analysis
(15)	679	Balloon-expandable (43%) and self-expandable (57%)	233 (34.3)	Increased all-cause mortality in LBBB group (26.6% vs. 17.5%; *p* = 0.006) Strongest predictive factors for all-cause mortality were: TAVI-induced LBBB (HR = 1.54; 95% CI = 1.12–2.10) and COPD (HR = 1.56; 95% CI = 1.15–2.10) A higher number of LBBB were observed after the implantation of a self-expandable valve (51.1% vs. 12%; *p* = 0.001)	Eight participating centers; ^†^

TAVI, Transcatheter Aortic Valve Implantation; LBBB, Left Bundle Branch Block; PPI, Permanent Pacemaker Implantation; EF, Ejection Fraction; NYHA, New York Heart Association; HR, Hazard Ratio; CI, Confidence Interval;

† *Patients with previous conduction disturbances or PPI were excluded from the analysis. When available, only predictors that persisted at multivariable analysis were reported*.

### Onset and self-resolution

Urena et al. analyzed a cohort of 202 patients undergoing TAVI with a balloon-expandable valve and with no previous conduction disturbance or PPI, and showed that of the 61 (30.2%) who developed LBBB during hospital stay, 85.2% recovered normal conductive function (59% at 7-day median discharge and 26.2% at long-term follow-up) (8); these findings were in concordance with other studies (6, 16, 17) and demonstrates that most of the new-onset LBBB are transient and do not require PPI implantation. In a cohort of 91 patients undergoing TAVI with self-expandable valve and with no exclusion of patients with previous conduction disturbances and/or PPI, Piazza et al. observed a higher incidence of 54% new-onset LBBB and of 45% at 6-month follow-up (7). These findings, corroborated by other studies (10), further suggest that self-expandable valves may cause a more severe mechanical injury to the conduction system as compared to balloon-expandable valves. Moreover, it was suggested that by not excluding patients with previous conduction disturbances and/or PPI, a higher rate of persistent LBBB might be observed (8).

### Impaired function recovery and reverse remodeling

Historically, the unfavorable effect of LBBB on systolic function is attributed to alterations in global and regional contraction and was proven both in otherwise normal subjects (18) and in hypertensive patients (19); furthermore, an adverse effect on diastolic function (19) and worse prognosis in comorbid patients (20) were also observed. Consequently, concerns were raised that in patients undergoing TAVI who develop persistent LBBB, a reduced EF recovery and therefore reduced benefits from the procedure might be observed.

Nazif et al. showed that in such cases a detrimental effect exists, with less or no EF recovery as compared to LBBB-free patients (58.1% vs. 52.8% at follow-up; *p* = 0.001) (2), independently of baseline EF. Carrabba et al. further elucidated that patients with new-onset LBBB lacked not only EF improvement, but also left ventricular remodeling (13). Urena et al. showed a decreased EF in patients with persistent LBBB at 1-year follow-up (Δ = 4.75 ± 8.02%, *p* = 0.031) (8), and Tzikas et al. reported similar findings also in patients treated with self-expandable valves (14). In another study by Urena et al., the only predictors of a lack of EF recovery were higher baseline EF and new onset LBBB (6).

### Impact on survival and functional class

There was no evidence of an impact of new-onset LBBB on patients survival after-TAVI (2, 6, 8, 13, 21) in all but one study by Houthuizen et al., which included patients with high logistic EuroSCORE (21%), therefore more prone to higher mortality rate (28.3%), regardless of whether the new-onset LBBB resolved spontaneously or not. No impact on rehospitalization was observed at 1-year follow-up (6, 8, 21) and no sudden death was reported in patients with new-onset LBBB and no PPI (8). The lack of increased mortality persisted also after a landmark analysis at 30-days (6).

Nonetheless, a poorer New York Heart Association class was observed at follow-up (18% vs. 7% in class II or higher, *p* = 0.015) (6, 8). Testa et al. failed to prove such a difference, although, when considering the high PPI rate in LBBB-free group (17 vs. 18%), it might be attributable to a worse-than-normal mechanical function also in the LBBB-free group (21). Therefore, in patients with persistent LBBB after TAVI, a strategy of early resynchronization seems reasonable, especially in patients with reduced LVEF.

Finally, new-onset persistent LBBB was also associated with an increased risk of AVB and need of PPI at follow-up (13.9 vs. 3.0%, *p* = 0.001, median time to PPI: 12 months) (6, 8). Although further studies are needed in order to confirm these findings, in this setting it might be reasonable to implement a strategy of close (24–48 h) ECG monitoring during the first months after TAVI or after systematic electrophysiology study (8).

### Predictors of left-bundle branch block after TAVI

Common limitations of studies investigating this topic are the inclusion of patients with pre-TAVI conduction disturbances and not taking in due consideration of the role of self- vs. balloon-expandable valves (3, 7), which led to controversial results in the past (6) (Table 3). When all these factors were taken into account, predictors of new-onset persistent LBBB were ventricular depth of the prosthesis (odds ratio [OR] = 1.37 for each increase of 1 mm) and baseline QRS duration (OR = 1.24 for each increase of 4 ms) (8); no predictors of transient LBBB were found (8) (Table 4).

**Table 3:** Rate of advanced conduction disturbances requiring PPI.

**Valve type**	**References**	**Design of the study**	**FU lenght**	**N**	**Rate of PPI**	**Comments**
Evolut R	(22)	Retrospetive, multi-center	30-days	120	21.9% (*n* = 19)	Only Evolut R 34 mm included
	(23)	Retrospective, sigle center	1-year	188	25% (*n* = 29)	Only 3 patients had AVB, the remaining had a prophylactic PPI
	(24)	Prospective, multi-center	1-year	1,038	19.3% (*n* = 175)	Unknown rate of AVB
	(25)	Prospective, multi-center	30-days	241	16.4% (*n* = 39)	Unknown rate of AVB
Sapien 3	(26)	Prospective, multi-center	1-year	1,946	11.5% (*n* = 195)	Unknown rate of AVB
	(27)	Propensity-matched cohort	1-year	622	15.5% (*n* = 87)	Cohort compared with ACURATE Neo, higher PPI implant in the Sapien 3 group
	(28)	Randomized trial	1-year	583	16.8% (*n* = 96)	PPI was required in 14.5% of HR patients and in 21.3% of inoperable patients
	(29)	Randomized trial	1-year	1,067	12.4%	Sapien 3 implant in intermediate-risk cohort
Lotus	(30)	Prospective, multi-center	1 year	1,041	34.6%	30.7% of PPI at 30 days, 3.9% after 30 days
	(30)	Multicenter, prospective	1 year	250	36% (*n* = 81)	Cohort of high-risk patients, unknown rate of AVB.
ACURATE neo Symetis	(27)	Propensity-matched cohort	1-year	311	9.9% (*n* = 28)	Cohort compared with Sapien 3, higher PPI implant in the Sapien 3 group
	(31)	Prospective, multicenter	1-year	1,000	8.3% (*n* = 83)	Unknown rate of AVB.

*PPI, Permanent Pamaceker Implant; AVB, advanced atrio-ventricular block; HR, high risk*.

**Table 4:** Predictors of conduction disturbances, pacemaker implantation and dependency after TAVI.

**Pre-procedural**	**References**	**Intra-procedural**	**References**
**PREDICTORS OF LBBB**			
Baseline QRS duration	(4)	Depth of prostheisis implantation	(4)
**PREDICTORS OF AV BLOCK**			
Male sex	(32)	New LBBB or RBBB	(32)
Short membranous septum	(33)	QRS > 128 ms	(34)
		Insufficient difference between membranous septum lenght and depth of implantation	
**PREDICTORS OF PPI**			
Male sex	(35)	New heart block	(35)
1st degree AV block	(35)	Self-expandable valve (vs. balloon-expandable)	(35)
Left anterior hemiblock	(35)	Depth of prosthesis implantation	(36)
Right bundle branch block	(35)	Valve oversizing	(37, 38)
Calcifications (aortic valve, LVOT, mitral valve, membranous septum)	(33, 39, 40)	Insufficient difference between membranous septum lenght and depth of implantation	(33)
**PREDICTORS OF PACEMAKER DEPENDENCY**			
Baseline LBBB	(41)	PR change after TAVI	(41)
PR duration before TAVI	(41)		
Porcelain Aorta	(41)		

While a longer QRS duration may be related to baseline conduction system damage and increased vulnerability (8), increased risk of new onset LBBB with lower valve implantation might reflect a more permanent damage to the conduction system with a more ventricular positioning (6). Moreover, this risk factor is consistent also when self-expandable valves are considered (42–44), suggesting that it might be intrinsic of the TAVI procedure.

## Advanced conduction disturbances after TAVI and PPI

A high rate of new AV and intraventricular conduction delays is observed within the first 48 h of TAVI, with a significant resolution by 30 days. About 22% of patients undergoing TAVI develop a post-operative new-onset AV block after balloon valvuloplasty or after valve deployment. These patients have a 5-fold higher risk of permanent AV block requiring a PPI (45). However, most of the complete AV block as well as the new-onset LBBB and AV blocks tends to disappear within the first days after TAVI: in a cohort of patients implanted with CoreValve, 19.7% had an absolute indication to PPI secondary to the development of advanced II degree AV-block and/or III degree AV block; however half of the advanced conduction delays resolved beyond the periprocedural period, waiting for more than 24 h following TAVI (46).

### Incidence of PPI after TAVI

The overall rate of PPI after TAVI ranges from 2 to 51% in a meta-analysis including 41 studies. The rate of PPI implant was 5 times more frequent in patients receiving a self-expandable Medtronic CoreValve (25–28%) compared to those who received a balloon-expandable Edwards Sapien/Sapien XT valve (5–7%) (47).

This increased risk of PPI with the CoreValve system was confirmed in the CHOICE randomized trial (Comparison of Transcatheter Heart Valves in High Risk Patients With Severe Aortic Stenosis), in which the rate of new PPI in the CoreValve group was 38% while in the Sapien XT group was 23.4% (*p* = 0.001) (48). The SURTAVI trial also confirmed high rates of PPI with both old generation CoreValve (25.5%) and new generation Evolut R (26.7%), despite the inclusion of intermediate-risk patients (49).

Focusing only on the latest-generation transcatheter heart valves, the incidence of PPI ranged between 2.3 and 36.1%. For balloon-expandable prostheses, the PPI rate was between 4.0 and 24.0% when using the new-generation Sapien 3 device, and a similar figure was observed with the previous generation Sapien XT device (ranging between 2.3 and 28.2%). For self-expandable prostheses, the PPI rates were higher with the early generation CoreValve device (16.3–37.7%), and despite a reduction in PPI rates with the new Evolut R, the rates remained relatively higher (14.7–26.7%) (50). These data are confirmed also in the latest experience with the new Evolut R device: among 1,038 patients, the rate of PPI was 17.8%. Similarly, the experience with the latest-generation Evolut PRO valve reports a rate of PPI of 11.8%; however, these results are limited by the low number of patients included in this early feasibility trial (*n* = 60) (51). A low incidence of PPI has been reported in case of Acurate *neo* implantation: in a recent large experience collected in 1,000 patients, the overall incidence of PPI was 8.3% (26); these data are confirmed in a recent propensity matched analysis comparing the Acurate *neo* and the Sapien 3: a high success rates was achieved for both valves, and the clinical and procedural results were comparable. However, Acurate *neo* required less frequently a PPI (9.9% vs. 15.5%; *p* = 0.02). Finally, the Lotus valve has been associated with higher rates of PPI than other devices (31.9–41.0%) (28, 52–54); this could partially be attributable to its peculiar design, including Adaptive Seal technology, which guarantees less paravalvular leak, but might poses a major risk toward the conduction system. Recently introduced strategies for higher implants (including the Lotus Edge Depth Guard Technology) might reduce the aforementioned stress on the conduction system and lead to lower PPI rates.

**Figure 2:**
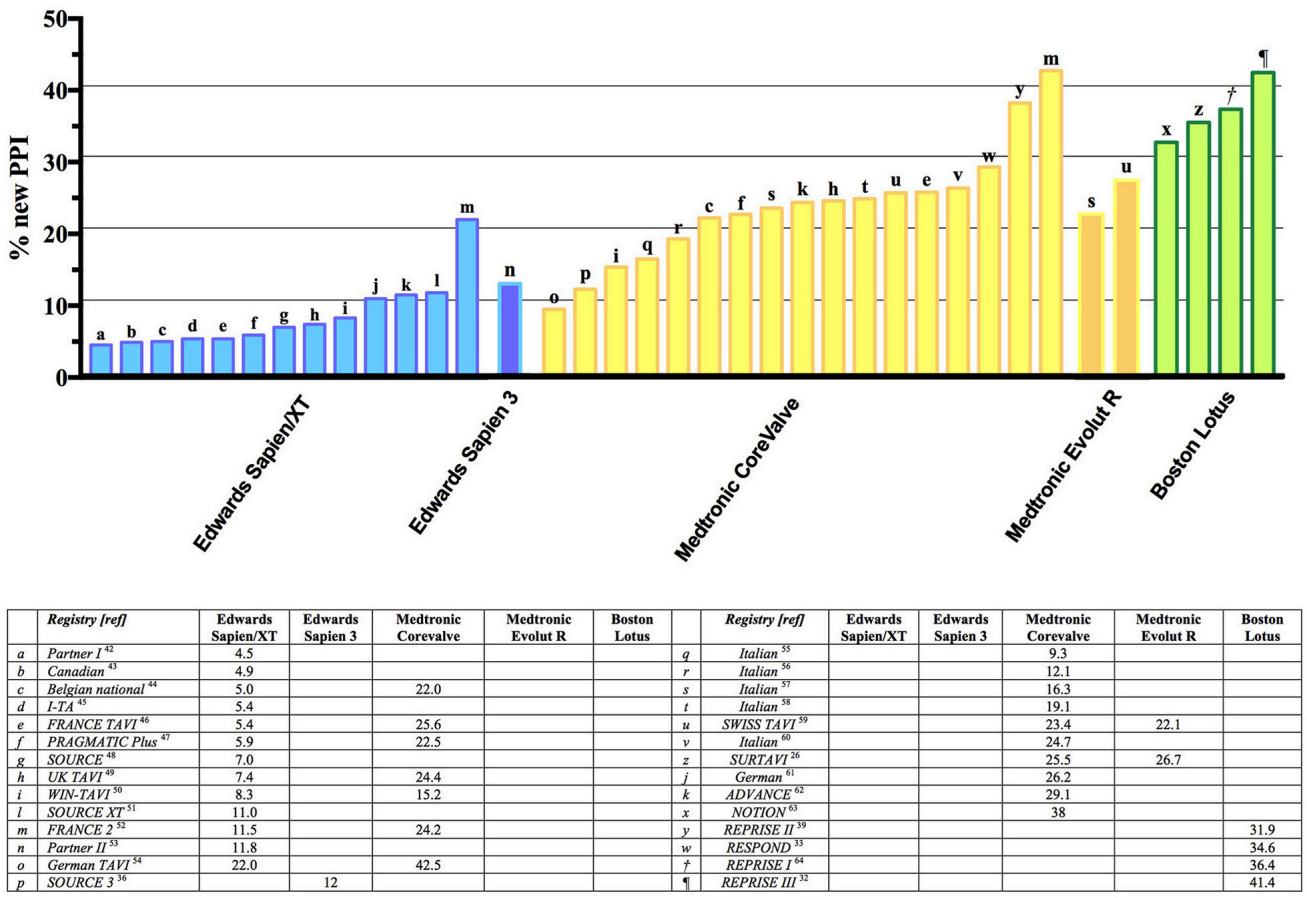
Summary of major trials and registries involving different types (both self-expandable and balloon-expandable) of valve and reporting incidence of new PPI. PPI, Permanent Pacemaker Implantation.

The prevalence of PPI among the most widely commercially available valves is reported in Figure 2. Although a clear trend can be observed, a huge variability in PPI was observed amongst different registries, even when the same valve was involved (Figure 2) (1, 22–25, 27, 29–31, 49, 51–75).

As reported by Auffret et al. (76), 2 main factors should be taken into account when evaluating the real incidence of PPI among different studies: first of all, indications to PPI are not uniform and do not always follow the canonic indication reported in the guidelines. As an example, some teams undertook prophylactic PPI in patients with new-onset LBBB after TAVR, which in turn resulted in an increased rate of PPI after TAVI. Moreover, the shorter period of observation after TAVI can underestimate the real incidence of PPI after the procedure. As demonstrated, a reduction in PPI rates has been observed with a strict adherence to Class I and II indications as recommended by clinical guidelines (12). Moreover, as experience, confidence and knowledge grows, a trend toward less PPI in single center registries has been observed (77).

As already mentioned, many of the newly developed advanced AV block resolves spontaneously, therefore according to the European Society of Cardiology guidelines, a prophylactic implantation of PPI after TAVI should be avoided and reserved only to those patients with recurrent AVB after an appropriate period of clinical observation with ECG monitoring (Class I, Level of Evidence C). Table 3 reports currently available data about the rate of advanced conduction disturbances requiring PPI.

Finally, the real incidence of PPI can be altered in some studies where patients with prior implant of PPI were included in the denominator, although not being exposed to the risk of new PPI implant.

Although guidelines remain vague and clear indications for PPI are still missing, many multicenter and literature-based decisional algorithms exist. In a recent state-of-the-art review, Auffret et al. proposed (76):

- ECG continuous monitoring until discharge for all patients who undergo TAVI;
- Same day PPI in all patients with a class I/II indication for PPI before TAVI;
- Temporary pacemaker for 24 h if new-onset LBBB and up to 48 h if new advanced AVB;
- PPI if new-onset LBBB persists 48 h after TAVI and QRS duration > 160 msec; consider loop recorder and/or electrophysiological studies and/or 30 days ECG monitoring in all other cases;
- PPI if advanced AV block persists 48 h after TAVI or recur before discharge (28, 54, 55, 57, 58, 78–82).

### Predictors of PPI after TAVI

In a recent meta-analysis of 41 studies including 11,210 TAVI recipients, male sex, first-degree AV block, left anterior hemiblock, and right bundle-branch block (RBBB) were identified as pre-procedural predictors of PPI, whereas the presence of intraoperative heart block and the use of a self-expandable prosthesis were the procedural predictors (35). In that study, the implantation of a CoreValve system was associated with a 2.5-fold higher risk of PPI, which was confirmed in another systematic review and in the recent report of the Society of Thoracic Surgeons Transcatheter Valve Therapy registry. Baseline RBBB is probably the strongest, most consistent clinical predictor of PPI; it has been identified in more than half of the studies evaluating multivariable predictors of PPI. Calcifications of the aortic valve (39), LVOT, and mitral annulus (40) and depth of prosthesis implantation (36) have been associated with PPI after TAVI. Proposed cut-off values for valve implantation depth predicting new-onset LBBB or PPI were 7 mm or 25% of the stent frame in the LVOT with the Sapien valve (37) and ranged from 6 to 7.8 mm with the CoreValve system (83) and from 5 to 6.7 mm with the Lotus valve (37). Values of 10 to 15% of valve oversizing have been associated with an increased risk of PPI with first-generation devices (37, 38). Concerning the post-procedural management of TAVI recipients, of particular interest are the predictors of delayed AVB after TAVI. In a larger series of 1,064 patients (45% with self-expandable valves), of whom 71 (6.7%) presented with delayed AVB (occurring 24 h after TAVI), Toggweiller et al. identified male sex and the presence of LBBB or RBBB after TAVI as independent predictors of delayed AVB (32). Mouillet et al. also proposed a post-TAVI QRS duration cutoff of >128 ms as a predictor of the evolution to AVB 24-h after TAVI (34). Baseline RBBB, PR interval duration before and after TAVI, PR interval change (>28 ms) within 3 days of TAVI, and porcelain aorta have been highlighted as independent predictors of pacemaker dependency at 1 year after TAVI (41). Finally, the membranous septum length, a surrogate for the distance between the aortic ring and the piercing bundle of His, has been proven as a major pre-intervention predictors of advanced AV block and PPI (33). In fact, mechanical compression of the emerging conduction tissue is easier if the membranous septum is too short and insufficient difference between this measure and the depth of implantation is achieved during TAVI.

### Prognostic impact of PPI after TAVI

Right ventricular apical pacing results in a left ventricular electrical activation sequence resembling left bundle-branch block. The resulting electrical asynchrony is manifest in a prolonged QRS duration due to slow myocardial conduction. Consequently, left ventricular contraction is altered, and significant interventricular and intraventricular dyssynchrony may occur (84) as result of a non-physiological activation. Ventricular desynchronization imposed by right ventricular apical pacing causes chronic left ventricular remodeling (85), including asymmetric hypertrophy and redistribution of cardiac mass, mitral regurgitation (86), increased left atrial diameter and reduced ejection fraction (87).

These adverse effects on ventricular structure and function likely explain the association of right ventricular pacing with increased risks of atrial fibrillation and heart failure in randomized clinical trials of pacemaker therapy. The MOST (Mode Selection Trial) demonstrated that heart failure during conventional cardiac pacing can be explained by complex interactions between substrate and promoters (11). Substrate is represented by clinical variables including atrial rhythm, AV conduction, ventricular conduction, ventricular function, symptomatic heart failure, and myocardial infarction. The promoters of heart failure are specific to the implementation of cardiac pacing and contain 2 constituents: ventricular desynchronization and AV desynchronization. Based on this model, patients with a very high-risk substrate (low ejection fraction, history of heart failure) are more likely to receive a negative impact from chronic right ventricular pacing (88).

The negative impact of PPI in TAVI patients has been largely explored in observational and retrospective studies (6). PPI after TAVI has been linked to and increased risk of recurrent hospitalizations for cardiovascular reasons and less recovery of left ventricular EF among patients with baseline impaired left ventricular function (89). In a meta-analysis published by Regueiro et al., the authors demonstrated a trend trough a reduction of cardiovascular deaths associated with the implantation of the PPI. The reason could be linked to the protective effect of pacing against the progression toward complete AV block and sudden death after TAVI. Conversely, the negative impact of PPI implant on mortality after TAVI was showed in a large patient population of 9,785 subjects. After multivariate adjustment, the authors found that PPI in TAVI patients was associated with a 31% increased risk for 1-year mortality and a 33% increased risk for a composite of mortality or heart failure admission at 1-year. Moreover, PPI was found to be associated with a prolonged length of stay in hospital (7 days vs. 6 days; *p* < 0.001) and in the intensive care unit (56.7 vs. 45.0 h; *p* < 0.001) (90). A smaller recent study of 1,973 patients from the PARTNER trial (91) and an international multicentre registry noted a trend toward increased 1-year mortality in patients with new PPI, but it did not reach statistical significance (92). Similarly, in a small study conducted on a cohort of patients treated with first-generation CoreValve, PPI was not associated with increased mortality at 1-year follow-up (93). Actually, only the large experience from the Society of Thoracic Surgeons/American College of Cardiology TVT registry demonstrated a negative influence of PPI on clinical outcome (90). Notably, PPI after TAVI has also been found to be protective against sudden death (92). The results of the most important studies on PPI and outcomes in TAVI patients are reported in Table 5 (89–94).

**Table 5:** Principal studies on PPI and outcome after TAVI.

**References**	**N**	**Type of valve**	**PPI recipients n, (%)**	**FU length**	**FU Mortality (PPI vs. no-PPI)**	**FU Hospitalization (PPI vs. no-PPI)**
(79)	1,347	SEV	*n* = 33.7%	30-days	NA	18.7 vs. 21.7% (*p* = 0.39)
(78)	275	SEV	*n* = 66 (24%)	1-year	12.5% vs. 11.8% (*p* = 0.9)	NA
(80)	2,559	BEV	*n* = 173 (8.8%)	1-year	7.6 vs. 9.0% (*p* = 0.52)	23.9 vs. 18.2% (*p* = 0.05)
(81)	9,785	BEV, SEV	*n* = 651 (6.7%)	1-year	24.1 vs. 19.6% (*p* = 0.003)	37.3 vs. 28.5% (*p* = 0.162)
(77)	1,556	BEV, SEV	*n* = 239 (15.4%)	36 months	36.1 vs. 31.5% (*p* = 0.73)	9.6 vs. 6.2% (*p* = 0.25)
(89)	1,629	BEV,SEV	*n* = 322 (19.8%)	4-years	48.5 vs. 42.9% (*p* = 0.15)	59.6 vs. 51.9% (*p* = 0.011)

*SEV, self expanding valve, BEV, balloon expanding valve, PPI, permanent pacemaker implant, FU, follow-up, NA, not available*.

The heterogeneity of data regarding PPI after TAVI can be interpreted in the light of the following points:

1. The negative effects of chronic right ventricular pacing may be difficult to demonstrate in the sicker TAVI population with a reduced life expectancy. A longer follow-up period is necessary to demonstrate the detrimental effect of chronic pacing.
2. The negative impact of chronic pacing could have a prognostic importance mainly in patients with reduced left ventricular EF.
3. The impact of right apical pacing on left ventricular EF is dependent both on the percentage of pacing and on pacing modality (i.e., DDD vs. VVI). Only few patients after TAVI have evidence of pacemaker-dependency, so that the negative impact of PPI implant becomes hard to be demonstrated.
4. The negative effect of chronic pacing is counterbalanced by the protective effect that PPI has at follow-up after TAVI. Patients with baseline RBBB and those with long LBBB (QRS length >160 ms) are at higher risk of death after discharge probably due to the development of AVB (92, 95). In this setting, PPI should be protective against the risk of suddendeath.

## Future perspectives

As TAVI becomes a widespread technology, it is becoming a safe and valid alternative for the treatment of aortic stenosis also in patients at intermediate surgical risk. The development of new transcatheter valves has led to a reduction in significant perivalvular leaks, but with a milder impact in the rate of PPI after TAVI. One of the main challenges in the TAVI field will be the reduction of advanced conduction disturbances needing PPI. This goal could be achieved through a better understanding of the clinical and procedural factors implicated in the development of conduction disturbances after TAVI and through a careful monitoring of patients developing conduction delays in order to avoid futile PPI. In this context, further studies should investigate the optimal timing for PPI after TAVI and evaluate factors associated with the development and recovery of conduction disturbances. Moreover, considering the aforementioned difference in PPI amongst different devices, it is reasonable to expect advancements in technology that could minimize the need of PPI especially when TAVI will be expanded to low-risk patients.

## Author contributions

All the authors contributed to the manuscript production and in the final revision. AM, CM, MP, and OD structured the manuscript giving contribute to table, figures and text editing. AC, LT, and AL revisited the article implementing the final manuscript form.

### Conflict of interest statement

The authors declare that the research was conducted in the absence of any commercial or financial relationships that could be construed as a potential conflict of interest.

## Abbreviations

AV: Atrioventricular
AVB: Advanced Atrioventricular Block
EF: Ejection Fraction
LBBB: Left Bundle Branch Block
OR: Odds Ratio
PPI: Permanent Pacemaker Implantation
TAVI: Transcatheter Aortic Valve Implantation.
